# Multiple simultaneous small intestinal diverticular penetrations

**DOI:** 10.1093/jscr/rjaf659

**Published:** 2025-08-23

**Authors:** Masahide Miyata, Hirofumi Tazawa, Akihisa Saito, Kazuya Kuraoka, Hirotaka Tashiro

**Affiliations:** Department of Surgery, NHO Kure Medical Center and Chugoku Cancer Center, 3-1, Aoyama, Kure 737-0023, Hiroshima, Japan; Department of Surgery, NHO Kure Medical Center and Chugoku Cancer Center, 3-1, Aoyama, Kure 737-0023, Hiroshima, Japan; Department of Diagnostic Pathology, NHO Kure Medical Center and Chugoku Cancer Center, 3-1, Aoyama, Kure, 737-0023, Hiroshima, Japan; Department of Diagnostic Pathology, NHO Kure Medical Center and Chugoku Cancer Center, 3-1, Aoyama, Kure, 737-0023, Hiroshima, Japan; Department of Surgery, NHO Kure Medical Center and Chugoku Cancer Center, 3-1, Aoyama, Kure 737-0023, Hiroshima, Japan; Department of Gastroenterological and Transplant Surgery, Graduate School of Biochemical and Health Sciences, Hiroshima University, 1-2-3 Kasumi, Minami-ku, Hiroshima, 734-8553, Hiroshima, Japan

**Keywords:** small intestinal diverticulum, simultaneous penetrations, functional end-to-end anastomosis

## Abstract

Jejunal diverticulosis is a rare condition. Here, we report a rare case of multiple simultaneous small intestinal diverticular penetrations. An 82-year-old man was admitted with a chief complaint of slurred speech. The patient had fever and elevated C-reactive protein (CRP) levels. Incidentally, two abscesses with increased density in the surrounding fatty tissue were identified using computed tomography. An emergency surgery was performed. A series of diverticula were found on the mesenteric side, spanning the area of abscess formation. We performed a partial resection of the jejunum, including abscess formation, and functional end-to-end anastomosis. Pathological examination indicated two instances of acute pyogenic diverticulitis with penetration. No cases of multiple simultaneous diverticular penetrations have been reported. However two previous reports described that of perforations. Most reports involved partial resection of the jejunum, including the perforated or penetrated site. Here, we report a rare case of multiple simultaneous small intestinal diverticulum penetrations.

## Introduction

Jejunal diverticulosis is a rare intestinal pathology, with an incidence of 0.5%–1% [1]. Herein, we report a rare case of a patient who presented with two simultaneously penetrating small intestinal diverticula.

## Case report

**Figure 1:**
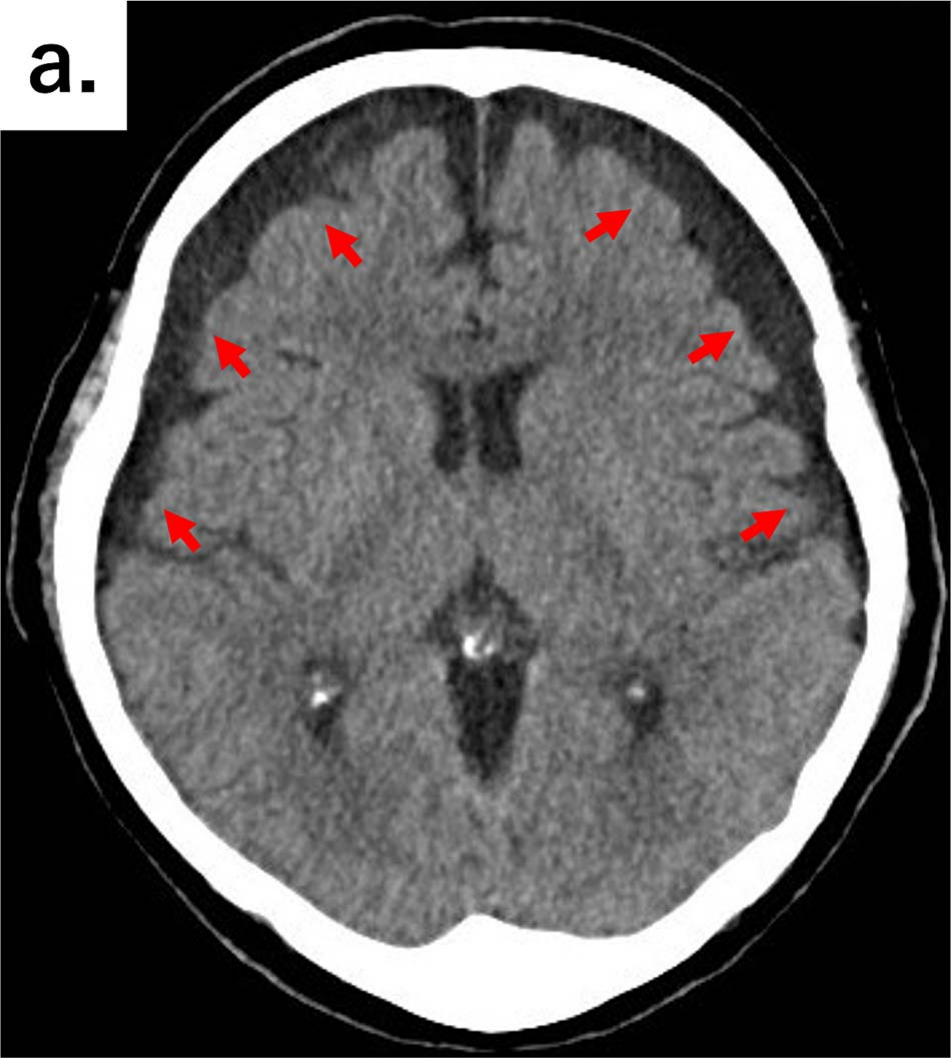
Axial CT image of the head showing bilateral chronic subdural hematomas (arrows).

**Figure 2:**
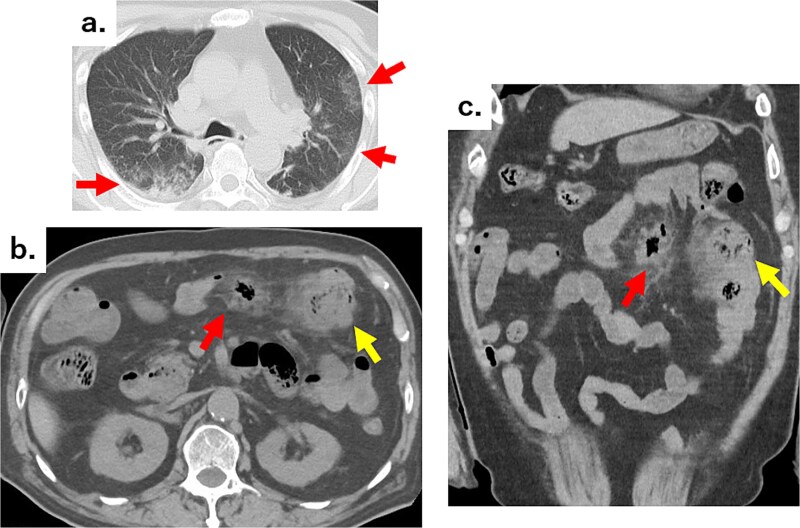
(a) Chest CT showing reticular shadows in both lung fields (arrows). (b) Axial and (c) coronal abdominal CT images showing scattered jejunal diverticula, abscesses in part of the mesentery (arrows), and an air formation with increased density in the surrounding fatty tissue (arrows).

An 82-year-old man visited the emergency department with a chief complaint of slurred speech. There were no digestive symptoms, such as diarrhea, malabsorption, bloating, chronic abdominal pain, or internal bleeding. His respiratory and circulatory dynamics were stable, and the blood test results were as follows: White blood cell (WBC) 12 200/mm^3^, Hgb 11.3 g/dl, blood urea nitrogen (BUN) 27.4 mg/dl, Cre 1.65 mg/dl, C-reactive protein (CRP) 7.79 mg/dl, no abnormalities in liver function, and no biliary system elevations. Computed tomography (CT) revealed chronic bilateral subdural hematomas (Fig. 1). Normal neck-to-pelvis CT revealed mild bilateral pneumonia (Fig. 2a). Moreover, multiple diverticula were found, primarily in the upper small intestine. Two abscesses showing increased density of the surrounding fatty tissue were identified: one was a 5-cm abscess in the mesentery of the small intestine and the other had air in the mesentery on the distal side (Fig. 2b and c). As there were no obvious signs of perforation and the patient's vitals were stable, conservative treatment was also an option, but the formation of a 5-cm abscess within the mesentery was a difficult location to puncture under CT guidance; therefore, we opted for emergency surgical treatment.

**Figure 3:**
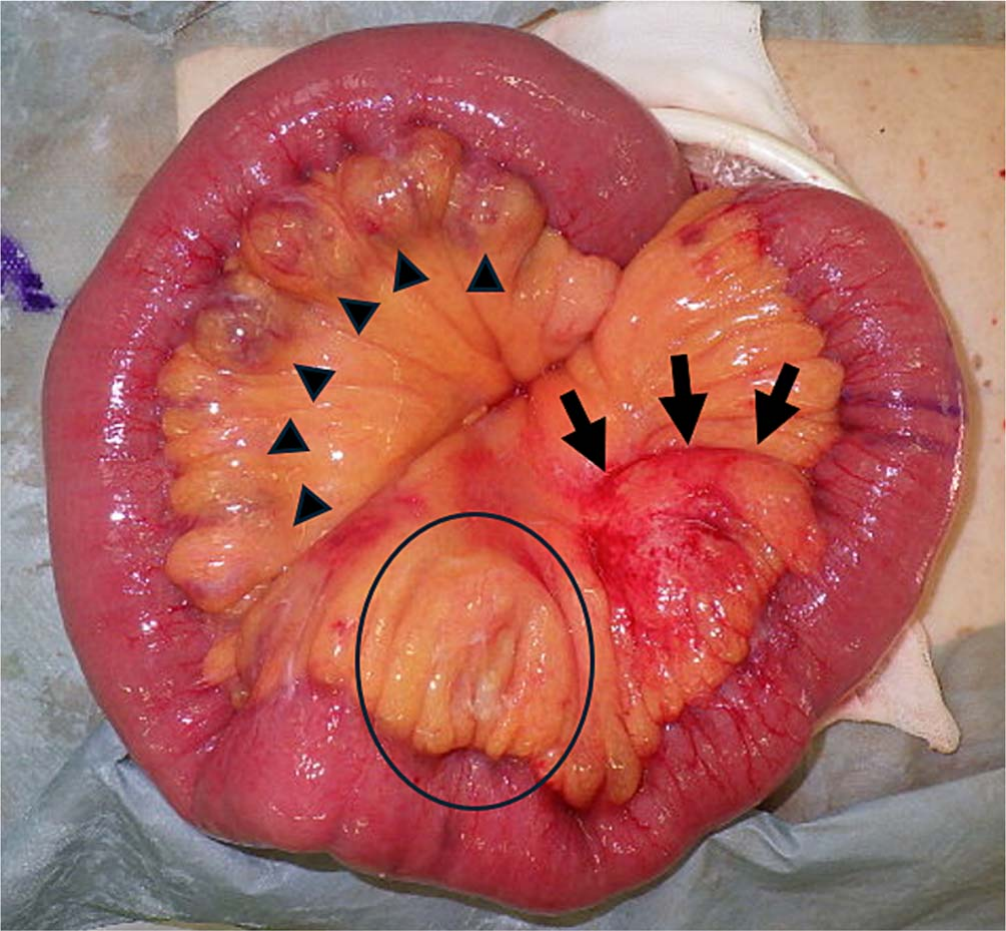
Macroscopic pathological specimen showing a series of diverticula measuring 2–5 cm in diameter, extending ~90 cm parallel to the mesentery of the jejunum, starting at the ligament of Treitz (triangles); a 5-cm mass with redness and hardness in the jejunal mesentery (arrows); and a change in hardness in the mesentery on the oral side (circle).

Emergency surgery was performed, beginning with an abdominal incision along the midline. The volume of ascites was small and there was no contamination. A hard mass approximately 5 cm in diameter was found in the jejunal mesentery, 60 cm from the ligament of Treitz. A series of diverticula were found on the mesenteric side, extending 90 cm further toward the anus from the ligament of Treitz and spanning the area of abscess formation (Fig. 3). We partially resected the small intestine, including the abscess site, without removing all the diverticula. We then performed an intestinal anastomosis using an automatic suturing device (functional end-to-end anastomosis: FEEA).

The patient started drinking water and taking medication the next day, and eating on the seventh day. The patient was discharged on Day 13 postoperatively.

**Figure 4:**
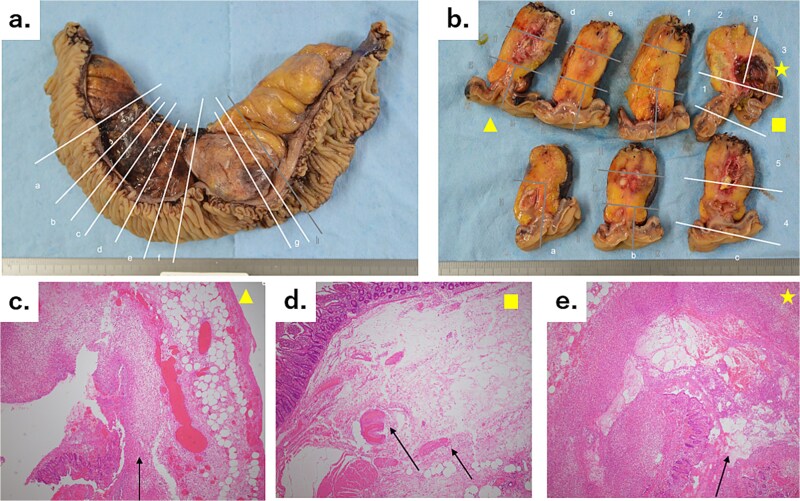
(a) A 30-cm small intestine segmental resection specimen showing a number of diverticula in the mesentery, a 5-cm mass with an abscess and bleeding, and an abscess in the mesentery on the oral side of the mass. The white lines marked with letters correspond to the slices shown in Fig. 4b. (b) Cross-sectional specimens showing scattered hard white nodules and abscess cavities. The two regions of inflammation are independent of each other, with no connections. The yellow triangle, square, and star correspond to Fig. 4c–e, respectively. (c) Microscopic image (hematoxylin–eosin staining, original magnification: ×4) showing the interrupted mucous membrane (arrow) and a collection of inflammatory cells, with bleeding in the surrounding area. (d) Microscopic image (hematoxylin–eosin staining, original magnification: ×4) showing the interrupted muscular layer (arrows). This diverticulum was a false diverticulum. (e) Microscopic image (hematoxylin–eosin staining, original magnification: ×4) showing the interrupted mucous membrane (arrow) and a collection of inflammatory cells, with bleeding in the surrounding area.

Pathological examination revealed a 5-cm mass and a hemorrhagic necrotic lesion in the mesentery of the small intestine segmental resection specimen. No apparent perforations were observed (Fig. 4a and b). In these two lesions, hematoxylin and eosin staining revealed discontinuity in the diverticulum wall surrounded by a cluster of inflammatory cells (Fig. 4c-e). Both lesions were diagnosed as acute pyogenic diverticulitis with penetration.

## Discussion

Most cases of jejunal-ileal diverticulosis are considered acquired diseases. This condition was first described by Summering in 1794, and subsequently by Sir Astley Cooper in 1807 [2]. No cases of multiple simultaneous diverticular penetrations have been reported, however two previous reports described that of perforations [3, 4]; the underlying mechanisms in both cases have not yet been elucidated. In this case, the patient had no history of inflammatory bowel disease, tuberculosis, or radiation exposure, which are known to predispose patients to small bowel perforation, or long-term steroid use.

Contrast-enhanced abdominal CT scans, which are the preferred imaging modality for complications of small intestinal diverticula [5], depict the location of the diverticulum and the surrounding tissue of the small intestine. Localized and asymmetric thickening of the small intestinal wall and inflammation or abscess of the periaortic fatty tissue are diagnostic criteria for diverticulitis of the small intestine. When these are combined with findings of extraintestinal gas, penetration or perforation can be diagnosed [6]. Perforation refers to a condition in which a hole forms in the intestinal wall, allowing intestinal fluid and free gas to leak into the abdominal cavity, leading to peritonitis. On the other hand, penetration refers to a condition in which a hole forms in the intestinal wall toward the mesentery, resulting in localized inflammation. In this case, CT scans did not reveal any perforation; however, blood tests showed signs of inflammation and an abscess that was difficult to drain. Therefore, we believe that the decision to perform surgery was correct.

**Figure 5:**
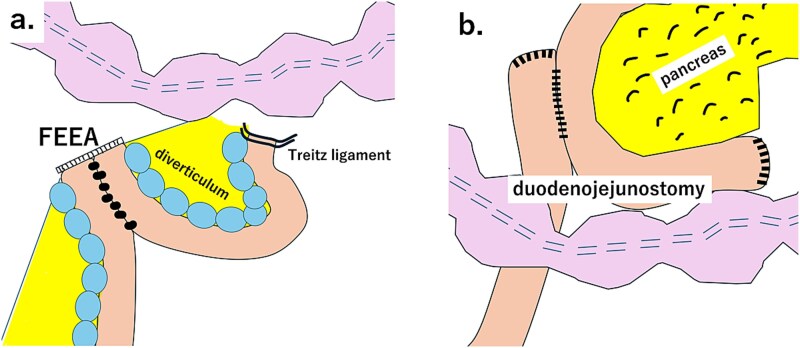
(a). Schematic diagram of partial resection of the small intestine and FEEA. (b) Schematic diagram of total resection of the small intestine diverticulum and duodenojejunostomy.

Regarding the extent of resection, we considered two options: One was partial resection of the penetrated area and anastomosis of the intestine (Fig. 5a), and the other was resection of the entire diverticulum and duodenojejunal anastomosis (Fig. 5b). In the second method, it requires dissecting and resecting the duodenum as closely as possible to the oral side of the ligament of Treitz. Using a retrocolic approach, it is necessary to perform a side-to-side anastomosis with the duodenum. In contrast, first method is less invasive than the second method; however, it necessitates resecting and anastomosing the jejunal diverticulum, which carries a higher risk of anastomotic leakage than in the second method. Because the diverticulum is located on the mesenteric side, anastomosis by the FEEA resulted in the anastomotic site being on the opposite side of the mesentery, thereby reducing the risk of anastomotic leakage. In addition, burying the staples at the end is preferable to prevent leakage. In this case, we performed a partial resection. FEEA was performed, and the anastomosis was completed on the contralateral side of the mesentery. Regarding the surgical procedure, most reports involve partial resection of the jejunum, including the perforated or penetrated site, abnormal findings, such as swelling of the mesentery, may be observed. Other reports include cases in which duodenojejunostomy was added because the perforation site was close to the ligament of Treitz [7], and cases in which the perforation was so small that only a wedge resection was performed [8]. However, wedge resection is only effective when a single diverticulum is located on the opposite side of the mesentery; this procedure is not possible for multiple diverticula on the mesenteric side, as in this case. Referring to previous reports, all cases occurred on the mesenteric side, and we believe that anastomosis of the contralateral side of the mesentery using FEEA was effective, as demonstrated in this case.

## Conclusion

Herein, we report a rare case of multiple simultaneous small intestinal diverticular penetrations.
